# Cytomegalovirus Proctitis Developed after COVID-19 Vaccine: A Case Report and Literature Review

**DOI:** 10.3390/vaccines10091417

**Published:** 2022-08-29

**Authors:** Yuqing Lv, Ying Chang

**Affiliations:** Department of Gastroenterology, Zhongnan Hospital of Wuhan University, Wuhan 430071, China

**Keywords:** COVID-19 vaccine, COVID-19, vaccine, CMV proctitis, cytomegalovirus, proctitis

## Abstract

(1) Background: We describe a case of a 58-year-old Chinese woman, without obvious cause of immunosuppression, who developed cytomegalovirus (CMV) proctitis three days after a second COVID-19 vaccination. Electronic colonoscopy revealed a new lesion that was circumferential at the anorectal junction, with an uneven surface and ulceration, which mimicked rectal carcinoma. This is the first case of CMV proctitis following vaccination since the invention of the COVID-19 vaccine, suggesting that the COVID-19 vaccine may cause disorders of immune homeostasis, including not only immune hyperactivity but also immune deficiency. We report this case to increase readers’ awareness of the risks after COVID-19 vaccination and to provide new ideas for the diagnosis and treatment of similar cases. (2) Methods: In this case, we used laboratory biochemical examinations, colonoscopy, immunohistochemistry, and a biochemical index to confirm the existence of CMV proctitis. (3) Results: In this case, the vaccine-induced CMV proctitis had a similar endoscopic appearance to rectal neoplastic lesions, which could be confirmed by biopsy and quickly relieved by ganciclovir treatment. Ganciclovir was used to treat the patient, and a good effect was observed. (4) Conclusions: COVID-19 vaccination may cause immune disorders, not just immune hyperactivity as previously reported, but also immune deficiency, such as CMV proctitis in this case. The clinical course of CMV proctitis secondary to COVID-19 vaccination was favorable with ganciclovir therapy.

## 1. Introduction

The COVID-19 epidemic is still raging over the globe, and vaccination is supposed to help us overcome it. The Chinese Center for Disease Control and Prevention (CCDC) has approved three forms of COVID-19 vaccines: inactivated vaccines (Sinopharm, Sinovac, etc.), recombinant vaccines (Chinese hamster ovary cells), and viral vector vaccines (adenovirus type 5 vector). Although the vaccinations’ efficacy is undeniable, their safety is still a concern. The advent of novel vaccines currently seems to be altering the course of events in a favorable direction. Along with the clear benefits stemming from the vaccination programs in many countries, side effects of these vaccines remain a concern that must be addressed. Hence, the recent reports of effective and safe vaccines were welcomed with great joy [1]. There have been a number of previous reports suggesting that COVID-19 vaccines may cause damage to multiple systems, most of which is caused by immune hyperactivity. A variety of mechanisms have been proposed to contribute to the rise in acute autoimmune responses [2]. As recently shown by Vorjani et al., molecular mimicry, i.e., antibodies against SARS-CoV-2 spike glycoproteins cross-reacting with structurally similar host peptide protein sequences, could play an important role in this response [3]. However, there are still some reports of immune-deficiency diseases such as herpes zoster virus and herpes simplex virus reactivation [4,5,6,7,8,9]. Although the mechanism of herpes zoster virus reactivation is elusive, the idea that some adjuvants in the vaccine may result in T-cell dysfunction is a reasonable guess. We reported a case of a 58-year-old Chinese woman, without obvious cause of immunosuppression, who developed persistent constipation three days after the second COVID-19 vaccination, and was eventually diagnosed with CMV proctitis that mimicked a rectal tumor under colonoscopy, which is the first case of post-vaccination immune-deficiency disease found in the digestive system. However, since its treatment with ganciclovir is highly effective, this should not distract us from the overwhelming benefits of COVID-19 vaccination.

## 2. Case Presentation

A 57-year-old Chinese woman suffered from constipation for several weeks after receiving her second dose of the CoronaVac COVID-19 vaccine. She was previously healthy. Further examination of the medical history revealed that the patient had difficulty defecating three days after the second injection of the COVID-19 vaccine in the community two weeks ago, accompanied by a feeling of perianal discomfort and abdominal distention. There was no obvious bloody stool or melena, and a colonoscopy at the local hospital indicated a chronic inflammatory reaction of the rectal mucosa. She denied any previous history of blood transfusion or anal intercourse. No diseases were recorded in her past medical history.

**Figure 1:**
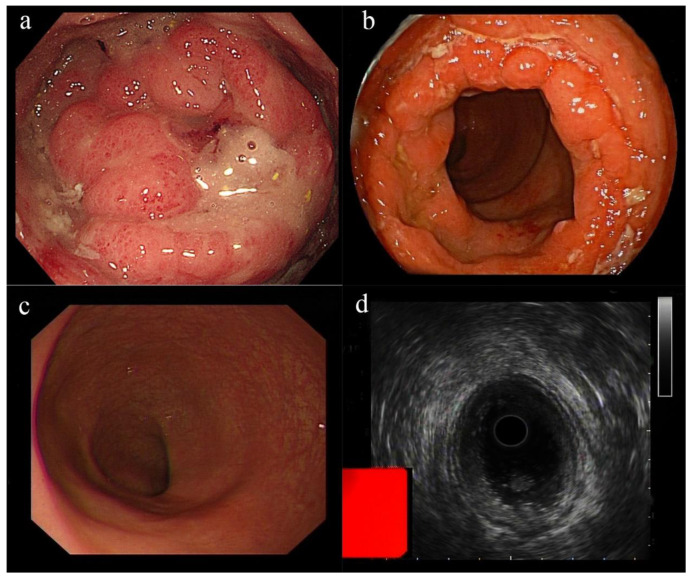
Proctosigmoidoscopy appearance: (**a**) A new lesion that mimicked rectal carcinoma at the anorectal junction. The surrounding mucosa was edemic and ulcerated. (**b**) Colonoscopy after treatment with ganciclovir showed shrinkage of the lesion. (**c**) A follow-up colonoscopy performed after 28 days of ganciclovir treatment revealed that the lesion had completely recovered. (**d**) Ultrasound colonoscopy revealed the lesion was confined to the mucosal layer, without extending beyond the mucosal muscular tissue, as the muscularis propria appeared to be intact.

A digital examination revealed a firm deformity over the posterior rectal wall. The ESR, CRP, and WBC were all within the normal limits. A stool routine examination showed a positive occult blood test (+). An anti-HIV and PCR test for COVID-19 were negative. We performed a colonoscopy on the second day of admission, revealing a new lesion that was circumferential at the anorectal junction, with an uneven surface and ulceration, which mimicked rectal carcinoma (Figure 1a). Further tests for tumor markers such as CEA, CA199, and CA125 were performed and found no abnormalities. Several biopsies were removed for further pathological examination. Pelvic enhanced rectal MRI revealed diffuse mucosal thickening in the middle and lower rectum, with limited diffusion of the contrast agent. Ultrasound colonoscopy showed that the lesion was confined to the mucosal layer, with intact mucosal muscular tissue and without invasion of the muscularis propria (Figure 1d). Subsequent liver MRI was performed to rule out the presence of tumor metastases, demonstrating a hepatic hemangioma of the lower right posterior lobe and liver cyst.

**Figure 2:**
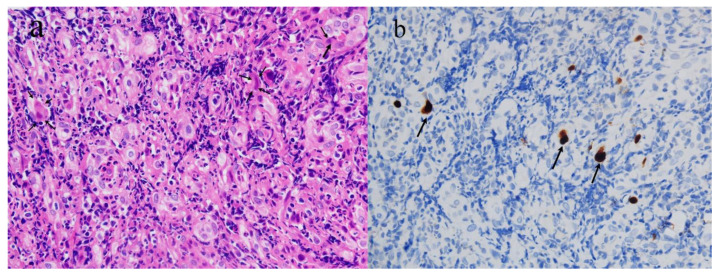
(**a**) Microscopic examination of rectal biopsy (×400, H&E stain). The large, infected cell showed an intranuclear viral inclusion with a perinuclear halo. (**b**) Immunohistochemical staining with monoclonal antibodies for CMV (×400) demonstrated positive uptake in inclusion bodies (arrow).

Two days after the colonoscopy, the biopsy revealed moderately active chronic proctitis with CMV infection (Figure 2). Microscopic findings showed a colorectal ulcer with fibrinoid necrotic debris, granulation tissue formation, and acute and chronic inflammatory cell infiltration. Intranuclear viral inclusions were seen in some infected cells. Immunohistochemical (IHC) staining with monoclonal antibodies for CMV confirmed CMV infections. CMV proctitis was diagnosed clinically.

The patient received 250 mg of ganciclovir intravenously twice daily for 5 days, and her intestinal discomfort, including abdominal pain and constipation, were completely resolved. A re-examination of the colonoscopy revealed that the rectal inflammation had subsided significantly, and the lesion had shrunk and no longer had a tumor-like appearance (Figure 1b). She was discharged in a stable condition after her symptoms resolved. After discharge, the patient was instructed to take 0.25 g ganciclovir capsules orally twice a day, one pill each time, reduced to one pill per day after one week. A follow-up colonoscopy performed after 28 days of ganciclovir treatment revealed that the lesion had completely recovered, with newly grown mucosal tissue, and a biopsy (Figure 1c) for CMV IHC staining was negative. Although we cannot prove whether the COVID-19 vaccine was the trigger of a new bout of CMV proctitis or whether it was just a contingent association, the brief duration of the symptoms and the timing of the COVID-19 vaccination administration led to the increased likelihood of the vaccine as a plausible culprit for her acute bout of constipation.

## 3. Discussion

There are several points of concern about this case: 1. This is a case of opportunistic disease caused by an immune disorder triggered by the injection of the COVID-19 vaccine. Previously reported diseases associated with COVID-19 vaccinations are usually attributed to immune hyperactivity, such as diseases of the digestive system [10,11,12,13,14,15,16]. The reactivation of herpes zoster virus in the skin has been reported as an immunodeficiency disease caused by immune disorders. Until now, no immune-deficiency diseases in the digestive system, such as CMV proctitis, had been reported. 2. The presentation of proctitis in this case was special, showing an appearance similar to that of a rectal tumor. We hope that this case will serve as a reminder to readers about whether proctitis following COVID-19 vaccination has a comparable appearance and performance, in order to avoid overdiagnosis and therapy. 3. CMV proctitis produced by the COVID-19 vaccine responds well to conservative medical treatment with ganciclovir, indicating that prompt identification and treatment do not hinder vaccination. The purpose of our report on this case is to enable readers to quickly identify, diagnose, and treat similar cases in a timely manner.

CMV disease rarely develops in immunocompetent patients, and reported cases often present with a mild, self-limiting course, without severe life-threatening sequelae [17]. According to a recent review of 290 cases of CMV disease in immunocompetent patients, the most frequently affected site was the gastrointestinal tract, but the rectum was rarely involved [17]. CMV proctitis can present in two distinct forms: primary and reactivated. It is pathognomonic for primary CMV proctitis for young patients to experience mononucleosis-like illness with rectal bleeding within several days to two weeks of unprotected anal intercourse. Reactivated CMV proctitis occurs mainly in elderly patients, without exposure to anal intercourse, who have poor underlying physical conditions, multiple comorbidities such as diabetes mellitus (DM), inflammatory bowel disease, and multiple organ failure, or who have immunodeficiency disease [18]. Obviously, in our case, the patient did not meet the criteria for primary CMV proctitis. Moreover, she was healthy, without a history of immune-deficiency disease, and was not receiving immunosuppressive drugs, which also contradicted the criteria for reactivated CMV proctitis. When the lesion involves the rectum, most patients present with diarrhea and tenesmus [19]. Meanwhile, in our report, the main symptom of the patient was constipation, because we observed a new tumor-like lesion under endoscopy. Although CMV proctitis with a tumor-like appearance has been reported before [20,21], it is still peculiar.

Due to their ability to reduce COVID-19-related disease’s severity and mortality [22], COVID-19 vaccines are being used worldwide. There have been some concerns regarding the possibility of COVID-19-vaccine-induced autoimmunity [23]. Indeed, antibodies against the spike protein S1 of SARS-CoV-2 have a high affinity for some human tissue proteins, suggesting that molecular mimicry may be responsible for this association [3]. Recently, some cases of multiple diseases that developed after COVID-19 vaccination have been reported, such as autoimmune hepatitis [10,11], myocarditis [24], thrombotic thrombocytopenia [25], and other diseases. There are some similarities between the previously described cases and the present case; one of them is a short interval between vaccination and symptom onset [11,26,27,28]. Most vaccine-related diseases are autoimmune, but there are still some reports of immune-deficiency diseases such as herpes zoster virus and herpes simplex virus reactivation (Table 1) [4,5,6,7,8,9]. Although a definitive theoretical elucidation of the underlying causes for herpes zoster virus reactivation remains elusive, there are still some conjectures. Psichogiou et al. [9] theorize that, following vaccination, a massive shift in the host’s T-cell response following vaccination may render the host’s zoster virus-specific adaptive immune cells momentarily incapable of managing latent zoster virus infection, which could be the underlying mechanism of virus reactivation. W.-H. Wang et al. [6] thought that it was possible that the vaccine caused certain immunomodulation that allowed the virus to awaken from latency. A review of 40 cases in an International Dermatology Registry reported 40 cases of varicella-zoster virus (VZV) and herpes simplex virus (HSV) reactivation, most of which occurred after the first vaccine dose [8]. Fathy, R.A. et al. [8] thought that herpesvirus reactivation may occur due to innate or cell-mediated immune-defense failures initiated by the host response to vaccination, although the precise mechanism is not known.

A literature search on PubMed for reports of cases following COVID-19 vaccination using the search terms (“COVID-19 vaccin*” [Title]) AND (case reports [Filter]) retrieved a total of 554 articles. The reports included in our review were limited to those written in English. The reference lists of relevant articles retrieved by the searches were also reviewed. We then screened out six COVID-19-vaccine-related diseases of the digestive system, as shown in the table below (Table 1).

As seen above, the majority of COVID-19-vaccine-related digestive diseases are autoimmune diseases due to immune hyperactivity. However, in our report, this is the first case of CMV proctitis in the digestive system following vaccination with the COVID-19 vaccine, further demonstrating that the COVID-19 vaccine may cause immune disorders rather than immune hyperactivity. A search of the Vaccine Adverse Event Reporting System (VAERS) database on 27 February 2022 yielded no events associated with COVID-19 vaccination and cytomegalovirus infection, suggesting this is a very interesting and rare case. VAERS accepts reports of adverse events and reactions that occur following vaccination. Most of the reports to VAERS are voluntary, which means they are subject to biases, whereas, to some extent, it proves that vaccine-related CMV infections are rare. 

However, there have been some recent reports of COVID-19-related CMV proctitis [30,31,32]. Maillet, F. et al. [30] described a case of reactivated biopsy-proven cytomegalovirus proctitis complicating the course of severe COVID-19 pneumonia treated with dexamethasone, anakinra, and lopinavir/ritonavir. Treatment with ganciclovir led to a favorable outcome. They theorize that the dysregulation of the immune system during COVID-19 pneumonia may cause a specific decrease in CD4+ T cells as in HIV patients, which leads to the development of CMV proctitis. On the other hand, some immunosuppressive agents used to combat COVID-19 inflammatory cytokine storm syndrome may lead to potential infectious adverse events. On the other hand, Plüß, M. et al. [29] reported a case of CMV reactivation after SARS-CoV-2 vaccination, which was thought to have a mechanism similar to immune reconstitution inflammatory syndrome (IRIS) because vaccination is a strong stimulator of the immune system. Therefore, we speculated that, in our case, the vaccine-induced CMV proctitis might have been related to abnormal lymphocyte function caused by immune-homeostasis disruption.

To sum up, we report a case of a 58-year-old Chinese woman who was previously healthy and had no underlying ailments, and developed CMV proctitis after receiving a second dose of the COVID-19 vaccine, which usually occurs in immunocompromised populations. A vaccine-induced immune disorder remains a highly suspected possibility, which may be confirmed by more similar examples.

## 4. Conclusions

The outcomes of this case scenario confirm CMV proctitis as a probable complication of COVID-19 vaccines. The clinical course was favorable with ganciclovir therapy. Immune disorders are more likely than immune hyperactivity to be involved in vaccine-related disease processes. Nevertheless, it should not distract healthcare providers from the overwhelming benefits of mass COVID-19 vaccination.

## Figures and Tables

**Table 1:** Summary of some reported cases of COVID-19-vaccine-related diseases.

Author	Patient’s Age/Sex	Past Medical Condition	Diagnosis	The Interval between Vaccination and First Symptom	Presenting Symptoms	Confirmed Conditions	Treatment	Outcome
Garrido et al. [10]	A 65-year-old woman	None	Autoimmune hepatitis	Two weeks after the first dose of Moderna COVID-19 vaccine	Mild abdominal pain, jaundice, and choluria	Liver histology showed a marked expansion of the portal tracts, severe interface hepatitis, and multiple confluent foci of lobular necrosis. Abdominal Doppler ultrasound showed hepatomegaly. Liver enzyme index increased	Treatment with prednisolone at 60 mg/day and a tapering course of corticosteroids.	Cure
Bril et al. [11]	A 35-year-old Caucasian female	Gestational hypertension	Autoimmune hepatitis	One 1 week after receiving her first dose of Pfizer–BioNTech COVID-19 vaccine	Generalized pruritus, choluria, and jaundice	Histology revealed the presence of eosinophils. Laboratories were significant for AST 754 U/L and ALT 2001 U/L. Doppler reported hepatomegaly without cirrhotic morphology	Treatment with prednisone at 20 mg daily	Cure
Cieślewicz et al. [12]	A 29-year-old female Caucasian	None	Pancreatic Injury	Twelve hours after the first dose of Pfizer–BioNTech COVID-19 mRNA vaccination	Muscle pain, headache, chills, and general weakness	Biochemical analysis revealed significantly increased CRP and urine amylase at 544 U/L. Magnetic resonance imaging of the abdomen suggested a mild pancreatic injury	The patient received paracetamol at 1 g i.v., a strict diet of fluids, gastroresistant capsules of pancreatic enzymes, and proton pump inhibitors	Cure
Parkash et al. [13]	A 96-year-old Caucasian female	Diastolic congestive heart failure, hypertension, hypothyroidism, cholecystectomy, and appendectomy	Acute pancreatitis	A few days after getting the first dose of Pfizer–BioNTech COVID-19 vaccine	Acute onset, severe abdominal pain	Her lipase level was significantly elevated, at 4036 U/L	She was monitored overnight with conservative treatment	Cure
Torrente et al. [14]	A 46-year-old Caucasian woman	Hypothyroidism and chronic iron deficiency anemia	Autoimmune hepatitis	3 weeks after the first Vaxzevria COVID-19 vaccination	Asymptomatic	Hypertransaminasemia. Laboratories showed AST 241 U/L, ALT 353 U/L, and GGT 44 U/L. Liver biopsy showed lymphoplasmacytic portal infiltrate with focal disruption of the limiting plate	Prednisone was initiated at a dose of 30 mg daily with a rapid improvement after 2 weeks of treatment, and azathioprine was added to treatment at a dose of 50 mg daily.	Cure
Hines et al. [15]	A 26-year-old woman	Irregular menses on oral contraceptives	ITP and acute liver injury	2 weeks after receiving the Moderna mRNA-1273 SARS-CoV-2 vaccine	Petechial rash	The peripheral blood smear showed rare schistocytes, and giant platelets, with her AST and ALT levels peaking on hospital day 3 at 446 U/L and 1257 U/L	Oral prednisone at 40 mg/day for 3 days. Dexamethasone at 40 mg IVP for 4 days. IVIG at 1 g/kg for 2 days.	Cure
Lensen et al. [1]	An 82-year-old woman	Alzheimer’s disease, HBV infection, HCV infection, DM, essential hypertension, osteoarthritis, portal hypertension with esophageal varices, and hepatic cirrhosis with thrombocytopenia	Hepatitis C virus reactivation	3 days after COVID-19 using Pfizer–BioNTech COVID-19 vaccine (first dose)	Jaundice, loss of consciousness, hepatic coma, and death	Hepatitis C PCR and hepatitis C antibodies were positive	Patient refused treatment with hepatitis C medication	Dead
Eid, E. et al. [4]	A 79-year-old man	Hypertension, coronary artery disease, and antineutrophilic cytoplasmic antibody-related glomerulonephritis	Herpes zoster	6 days after receiving the mRNA COVID-19 vaccine	Itchy and tender lesions over the right thigh	On dermatologic examination, a confluence of vesicles, some excoriated and overlying an erythematous base, were appreciated scattered over the right thigh in a dermatomal distribution	Systemic antiviral treatment	Cure
David, E. et al. [5]	A 41-year-old woman	A history of varicella infection in childhood	Herpes zoster	3 days after vaccination with Moderna COVID-19	Fatigue and left arm soreness around the injection site, diarrhea, skin pain affecting the left lower back, and vesicular rash	Physical examination revealed a cluster of pink to red erythematous urticarial appearing papules and plaques with overlying clustered vesicles. Vesicular fluid was collected for VZV DNA PCR, which yielded a positive result.	Without treatment	Cure
Chiu, H.H. et al. [6]	A 71-year-old man	None	Herpes zoster	2 days after his first injection of Moderna COVID-19 vaccine	grouped erythematous papules and vesicles appeared on his left flank with itching and pain	Based on clinical manifestations, HZ involving left T8 dermatome was diagnosed	Oral acyclovir for 1 week	Cure
	A 46-year-old man	None	Herpes zoster	2 days following receiving his first dose of AZD1222 vaccine	Pain and itch over ipsilateral flank	HZ was diagnosed later when the typical clinical presentation of HZ as grouped vesicles was present over left T11	Oral acyclovir for 1 week	Cure
Kerr, C. et al. [7]	A 71-year-old woman	Immunoglobulin A nephritis, and a history of chickenpox in childhood	Varicella-zoster virus meningitis	1 day after her first BNT162b2 mRNA COVID-19 vaccination	Fever and headache	A final diagnosis of VZV meningitis was made based on positive rapid immunochromatography (abdominal vesicles tested) and CSF polymerase chain reaction (PCR) results (1.86 × 10^6^/μL)	Intravenous acyclovir treatment	Cure
Plüß, M. et al. [29]	A 67-year-old Caucasian female	Atrial fibrillation, hypertension, obesity, degenerative knee joint disease, and no documented history of COVID-19	Cytomegalovirus reactivation and pericarditis	Two weeks after first dose of ChAdOx1 nCoV-19 vaccination	She suffered from fever, weakness, and arthralgia of the knees, hips, and shoulders	Cardiac magnetic resonance imaging (MRI) confirmed diagnosis of pericarditis with circumferential thickening and contrast enhancement of the entire pericardium at late gadolinium enhancement. CMV infection was confirmed by PCR with detectable CMV viremia	Oral valganciclovir was initiated (900 mg twice daily) for three weeks	Cure
Our report	A 58-year-old Chinese woman	None	CMV proctitis	Three days after the second dose of CoronaVac COVID-19 vaccine	Constipation, perianal discomfort, and abdominal distention	Proctosigmoidoscopy revealed new circumferential growth at the anorectal junction, with an uneven surface and ulceration. A biopsy revealed moderately active chronic proctitis with CMV infection	The patient received 250 mg of ganciclovir twice daily for 5 days, and oral ganciclovir was continued after discharge	Cure

COVID-19 = coronavirus disease 2019; CRP = C-reactive protein; IVP = intravenous drip; IVIG = intravenous immunoglobulin; PCR = polymerase chain reaction; SARS-CoV-2 = severe acute respiratory syndrome coronavirus 2; ITP = idiopathic thrombocytopenic purpura; CMV = cytomegalovirus; HZ = herpes zoster; VZV = varicella-zoster virus; CSF = cerebrospinal fluid.

## Data Availability

Not applicable.
